# Integration of Algae to Improve Nitrogenous Waste Management in Recirculating Aquaculture Systems: A Review

**DOI:** 10.3389/fbioe.2020.01004

**Published:** 2020-09-04

**Authors:** Norulhuda Mohamed Ramli, J. A. J. Verreth, Fatimah M. Yusoff, K. Nurulhuda, N. Nagao, Marc C. J. Verdegem

**Affiliations:** ^1^Aquaculture and Fisheries Group, Wageningen University & Research, Wageningen, Netherlands; ^2^Department of Biological and Agricultural Engineering, Faculty of Engineering, Universiti Putra Malaysia, Serdang, Malaysia; ^3^International Institute of Aquaculture and Aquatic Sciences, Universiti Putra Malaysia, Port Dickson, Malaysia; ^4^Department of Aquaculture, Faculty of Agriculture, Universiti Putra Malaysia, Serdang, Malaysia; ^5^Bluescientific Shinkamigoto Co. Ltd. (BSCIS), Nagasaki, Japan

**Keywords:** algal cultivation, nitrogen, recirculating aquaculture system, ammonia, nitrate, removal rates, HRT

## Abstract

This review investigates the performance and the feasibility of the integration of an algal reactor in recirculating aquaculture systems (RAS). The number of studies related to this topic is limited, despite the apparent benefit of algae that can assimilate part of the inorganic waste in RAS. We identified two major challenges related to algal integration in RAS: first, the practical feasibility for improving nitrogen removal performance by algae in RAS; second, the economic feasibility of integrating an algal reactor in RAS. The main factors that determine high algal nitrogen removal rates are light and hydraulic retention time (HRT). Besides these factors, nitrogen-loading rates and RAS configuration could be important to ensure algal performance in nitrogen removal. Since nitrogen removal rate by algae is determined by HRT, this will affect the size (area or volume) of the algal reactor due to the time required for nutrient uptake by algae and large surface area needed to capture enough light. Constraints related to design, space, light capture, and reactor management could incur additional cost for aquaculture production. However, the increased purification of RAS wastewater could reduce the cost of water discharge in places where this is subject to levees. We believe that an improved understanding of how to manage the algal reactor and technological advancement of culturing algae, such as improved algal reactor design and low-cost artificial light, will increase the practical and economic feasibility of algal integration in RAS, thus improving the potential of mass cultivation of algae in RAS.

## Introduction

According to FAO (2019), aquaculture production increased steadily at an average of 4.8% per year from 2010 until 2017. In 2017, aquaculture contributed 46.4% of the world total fishery production, which is equivalent to 80.1 million metric tons (valued at USD 23.8 billion). This share is expected to reach 52% in 2025, which is equivalent to 102 million tons of production. This indicated that the aquaculture sector will be the main driver of the world fish supply (FAO, 2019).

Fish production systems can be categorized into three types: flow-through systems (cages and raceways), semi flow-through systems (ponds), and recirculating aquaculture systems (RAS) where either nutrients, water, or both are recycled. The highest water exchange rate is applied in the flow-through system (>50 m^3^ kg^–1^ feed), medium exchange rate in the semi flow-through system (1–50 m^3^ kg^–1^ feed) and minimum exchange rate in the conventional RAS (0.1–3 m^3^ kg^–1^ feed) (Martins et al., 2010; Bregnballe, 2015). RAS has an advantage over the flow-through and semi flow-through systems because waste discharge into the environment can be controlled, a smaller volume of water per kilogram fish production is used, higher biosecurity standards can be applied, and in cases where antibiotics have to be applied, discharge to the environment can be prevented. Due to expected water scarcity, limited area for aquaculture (FAO, 2016), and increasingly strict environmental regulations, RAS has become more important for aquaculture activities.

Recirculating aquaculture systems are intensive systems, which rely on formulated feed to provide all the nutrient requirements for the cultured organisms. A RAS must consist of a self-cleaning-conditioning system, which the water is reused for culture (Timmons and Ebeling, 2007). Analyzing information in the literature, Schneider et al. (2005) concluded that between 50 and 70% of feed nitrogen (N) becomes waste in the culture system. Meanwhile, fish feeds usually contain a high percentage of crude protein, between 30 and 60%. According to Ebeling et al. (2006), when introducing 1 kg of feed containing 32% crude protein in a 1 m^3^ RAS, 30 g ammonia-nitrogen (ammonia-N) will be released, which in this case will raise the ammonia-N concentration by 30 mg L^–1^.

In a RAS, the concentration of ammonia-N must be maintained below 1 mg L^–1^ due to its harmful effect upon fishes (Greiner and Timmons, 1998; Timmons and Ebeling, 2007). Total ammonia nitrogen (TAN) consists of unionized ammonia (NH_3_) and ammonium ion (NH_4_^+^). In this article, ammonia and ammonium are referred to the unionized and ionized species, respectively. Meanwhile, total ammonia is referred to as TAN. The toxicity of NH_3_ is related to dissolved carbon dioxide (CO_2_) concentration and pH of the water. As dissolved CO_2_ decreases, the pH increases and increases the toxicity of NH_3_ (Timmons and Ebeling, 2007). Dissolved CO_2_ is continuously produced in RAS *via* fish and bacterial respiration, and bacterial decomposition. A degassing process is integrated into a RAS to control CO_2_ concentration. Meanwhile, pH of water in RAS could decrease as a result of the nitrification process; therefore, bicarbonate is added to regulate pH in the system. Failure to manage dissolved CO_2_, and pH could expose the fish to a higher risk of TAN toxicity. In nitrification, TAN is converted to nitrite and nitrite to nitrate. Nitrite is a toxic species of inorganic nitrogen at levels more than 1 mgL^–1^, and nitrate is found harmful for many fresh water fish species at concentrations above 1,000 mgL^–1^ and for marine species at concentration more than 500 mgL^–1^ (Colt, 2006).

In a RAS, recycling reduces the amount of water use needed. In order to maintain the water quality in a RAS while keeping water renewal limited, a series of water purifying units can be installed, such as a solids removal unit, a biological filtration unit for inorganic nitrogen removal, and a reservoir where water conditioning may take place (likes heating, oxygenation, and disinfection) (Bregnballe, 2015). The biological filtration unit controls the concentration of total ammonia. The key process for controlling the total ammonia level is autotrophic nitrification, which converts total ammonia into nitrite and nitrite into nitrate. However, the product of nitrification, nitrate, accumulates in the RAS. Therefore, in recent RAS configurations, a denitrification reactor often is added to maintain a low level of nitrate in the system. The concentration of nitrate-N (NO_3_-N) can be as high as 400 to 500 mg NO_3_-N L^–1^ in a conventional RAS without the denitrification component (Van Rijn et al., 2006). The high nitrate concentration can have adverse effects on the growth of farmed organisms (Davidson et al., 2017).

However, denitrification is not a productive process in the sense that the inorganic nitrate-N is converted to N_2_ gas, a non-readily useful form of nitrogen. At the same time, producing inorganic N fertilizers from N_2_ gas is an energy-intensive process (Bartels, 2008). Therefore, to improve the sustainability of a RAS, alternative approaches for ammonia, nitrite, and nitrate conversion need to be explored, such as assimilation of nitrogen by organisms that can be subsequently harvested. An example is assimilation by algae. Recent studies show many benefits of integrating algae in an aquaculture production system. They improve the stability of water quality of a RAS (Ramli, 2018) and may help to control harmful bacteria in the culture water (Defoirdt et al., 2004; Natrah et al., 2011, 2014; Tendencia et al., 2013), or remove heavy metals and organic contaminants from the water (Muñoz and Guieysse, 2006; Matamoros et al., 2015; Suresh Kumar et al., 2015). Integration of algae in a RAS could be relatively easy and inexpensive, as demonstrated in a study by Valeta and Verdegem (2015), which used an algal-turf-scrubber in a RAS. On the other hand, anaerobic denitrification units are expensive and can prove finicky to operate. This statement is based on the authors’ personal experiences working with an experimental setup of an up-flow sludge blanket manure denitrification reactor and from a study by Meriac et al. (2014a) that integrated a denitrification reactor in RAS.

Besides improving water quality, algae may be an important fish food because they contain high protein (between 40 and 70%), carbohydrate (between 10 and 65%) and lipid (between 5 and 45%) per unit dry weight and contain polyunsaturated fatty acids, such as docosahexaenoic acid (22:6 *n* − 3), eicosapentaenoic acid (20:5 *n* − 3) and arachidonic acid, which can be used by various fish and larvae (Sargent et al., 1997; Becker, 2013; Roy and Sen Pal, 2015). Recently the use of microalgae in fish feed has become more significant as microalgae can potentially reduce the need for inclusion of fish meal and fish oil in fish feeds (Shah et al., 2018). Neori (2011) reported that microalgae use as feed through the technique of green-water culture serves as an important driver to increase production of planktivorous species such as Nile tilapia (*Oreochromis niloticus*), rohu carp (*Labeo rohita*), bighead carp (*Hypophthalmichthys nobilis*), catla (*Catla catla*), and shrimps.

Many reviews have covered extensively the cultivation of algal in different environments such as open and closed microalgae production systems (Masojídek and Torzillo, 2008; Mata et al., 2010; Yusoff et al., 2019) and various wastewater systems (Larsdotter, 2006a; Pittman et al., 2011; Abdel-Raouf et al., 2012). These studies have demonstrated benefits of integration of algae in the systems for nitrogenous waste management. Works related to algae integration in RAS is limited, and no recent report was found except for studies by the authors (Ramli et al., 2018a,b) that deal with algae-bacteria interactions in a RAS. Van Rijn (2013) reported that integration of phototrophic organisms (such as algae) in a RAS was mainly restricted to outdoor RAS due to the large areas required for photosynthesis. Since the development has been rather slow, the state-of-the-art is limited to work presented in this review. Consequently, the potential and feasibility of integration of microalgae in a RAS, especially indoors, for nitrogenous waste removal remains unclear and must be explored. Therefore, the objective of this paper is to review nitrogen removal performance by algae subject to different configurations of RAS.

The review starts by indicating differences between RAS integrated with algae and integrated multi-trophic aquaculture (IMTA), which also integrates algae in the system (see Section “The Differences Between RAS Integrated With Algae and IMTA”). Next, this review examines how different RAS has exhibited different nitrogen removal rates by identifying variables or factors that might influence removal rates (see Section “Integrating RAS With Algal Reactor”). These identified factors are further discussed (see “Factors Affecting Nitrogen Removal Rates by Algae”). Some insights on cost-benefit analysis on integrating algae in RAS are discussed in Section “Cost-Benefit Analysis of RAS-Photobioreactor Integration.” Information gathered in this review will give better insights into how nitrogen removal by algae in RAS could be optimized and assess whether RAS could be used for mass cultivation of algae.

## The Differences Between RAS Integrated With Algae and IMTA

One of the aquaculture technologies which also uses algae for nitrogenous waste management is IMTA. According to Buck et al. (2018), in IMTA, fed species, mainly fish or shrimps, are co-cultivated with extractive species such as suspension-feeders (e.g., mussel or oyster), deposit-feeders (e.g., sea-cucumber or sea-urchin), and macroalgae (e.g., kelp). One of the main objectives of IMTA is to increase the productivity per unit of feed given to a system, thus increasing the sustainability of aquaculture activities (Neori et al., 2004). The concept of IMTA applies to coastal lagoons, bays, and inland aquaculture such as ponds and tanks. In ponds, the culture water could be partly or fully channeled to algae culture systems and recirculated, and thus can be considered as a RAS or semi-RAS for inland aquaculture (Neori et al., 2003, 2017; Schuenhoff et al., 2003; Abreu et al., 2011). However, in open systems such as coastal lagoons, sequestration of the wastes by algae, though promising, is difficult to measure due to dilution (Troell et al., 2003; Buck et al., 2018), and uncertainties in estimating the contribution of nutrients already present in the open production environment.

Recirculating aquaculture systems and IMTA were originally conceptually different in terms of nutrient recycling. RAS aims to maintain water quality for cultured species while removing waste products such as organic particles (sludge) coming from feces, remnant feed, and sloughed biofilm from biofilter media. In RAS, dissolved ammonia is not removed but converted into less toxic nitrate, while both ammonia and nitrate can also be taken up by algae. In addition, nitrogen can be removed through denitrification. In contrast, IMTA aims through co-cultivation of extractive species to trap as much nutrients as possible in commercially valuable species or products. As a result, in practice IMTA and RAS partially overlap. This review focuses on the integration of algae in RAS, not covering the nutrient trapping by algae and the fate of algae in open systems. Findings presented are, however, also relevant to land-based nearly-closed or semi-closed IMTA.

## Integrating RAS With Algal Reactor

An RAS must include of a series of water purifying units, namely a solids removal unit and a biofiltration unit. The concept of a RAS was originally designed for indoor systems (Rakocy et al., 1992), and this concept has been broadened to include outdoor pond systems (Lazur and Britt, 1997; Bosma and Verdegem, 2011). In this review, for the outdoor ponds to be considered a RAS, the fish culture pond must be associated with a water purification pond or other unit for biofiltration. Meanwhile, RAS also are operated in greenhouses, as demonstrated in the study of Huang et al. (2013). The greenhouse offers protection from the environment, is easily controlled, and uses mainly natural light. For this review, RAS in a greenhouse is considered as indoor RAS because of the protection it receives.

The main processes for water treatment in a RAS are solids separation and biological treatment processes mainly for transforming inorganic nitrogenous wastes into nitrate or nitrogen gas through nitrification/denitrification or for ammonia assimilation into algae and bacteria. In an outdoor RAS, the biological processes may occur simultaneously in ponds, whereas, in an indoor RAS, bacterial and algal processes typically are compartmentalized and managed specifically to support the purification process in each compartment.

This review focuses on a RAS, which has at least one algae tank or pond as a bio-filtration unit separated from the main culture unit be it outdoor or indoor. Currently, there is a limited number of studies on RAS integrated with algae (Table 1) (Further descriptions of these systems can be found in Supplementary Table 1).

**TABLE 1:** Rates of nitrogen removal by algae reactors in recirculating aquaculture systems (RAS).

System type	Cultured animal (stocking density, kg m^–3^)	Feeding	Nitrogen loading rate, g m^–2^ day^–1^	Nitrogen removal rate, g m^–2^ day^–1^	References
**Microalgae (indoor)**					
Valeta and Verdegem (2015) – Study 2 RAS + Periphyton turf scrubber, (PTS) which comprise microalgae and other micro-organisms	Tilapia (*Oreochromis niloticus* L) (28–70)	Commercial feed (CP was not given)	3.76–3.81 g TAN	3.302–0.656	Valeta and Verdegem, 2015
Huang et al. (2013) - Study 3 RAS + PTS	Rainbow mussels (*Villosa iris*) (245 animals m^–2^, 17.3 mm length)	*Nannochloropsis* (0.02 ml L^–1^) and mixture of *Isochrysis* sp., *Pavlova* sp., *Thalassiosira weissflogii*, and *Tetraselmis* sp. (0.007 ml L^–1^)	n.a	3.01	Huang et al., 2013
**Microalgae (outdoor)**					
SustainAqua (2009) – Study 4 RAS + outdoor PTS	Fishpond - common carp (*Cyprinus carpio*) (15) Periphyton pond-tilapia (*Oreochromis niloticus* L) (0.5)	Commercial feeding in fishpond (40% CP)	3.8	3	SustainAqua, 2009
Gál et al. (2003) – Study 7 Combined Intensive – extensive pond system	Extensive pond – 90% common carp (*Cyprinus carpio*) and 10% Chinese carp (*Hypophthalmichthys molitrix* and *Aristichthys nobilis*) (0.014–0.15) Intensive pond – common carp (*Cyprinus carpio*), African catfish (*Clarias gariepinus*) and tilapia (*Oreochromis niloticus*) (0.07–3.9)	Commercial feed (0.042–0.06 g N day^–1^) (CP was not given)	3.87	3.26	Gál et al., 2003
Li et al. (2019) – Study 8 RAS- outdoor raceway (microalgae, multi species)	European sea bass (*Dicentrarchus labrax* L.) (30- initial density)	Commercial feed (*ad libitum*) estimate CP = 43%	3.435	3.391	Li et al., 2019
Macroalgae (indoor)					
Cahill et al. (2010) – Study 1a RAS + Algae tank (*Ulva lactuca*)	Rainbow abalone (*Haliotis iris*) (4.45)	Commercial feeding (0.75% body weight) CP not mentioned, but 35% used from reference	3.1134^1^	*0.135	Cahill et al., 2010
Cahill et al. (2010) – Study 1b RAS + Algae tank (*Ulva pinnatifida*)	Rainbow abalone (*Haliotis iris*) (0.004)	Commercial feed (0.75% body weight)	*0.1134	*0.135	Cahill et al., 2010
**Macroalgae (outdoor)**					
Pagand et al. (2000) – Study 5 RAS + HRAP - Mix of microalgae and macroalgae species (*Ulva* sp., *Enteromorpha* sp. *Ectocarpus* sp.)	European sea bass (*Dicentrarchus labrax* L.) (100)	Commercial feed (n.a) estimate CP = 40%	3.6	3.9 (winter) 2.79 (summer) (0.5 to winter; 0.9 summer) (0.1 g N g^–1^ algae day^–1^)	Pagand et al., 2000
Deviller et al. (2004) – Study 6 RAS + HRAP – Mix of microalgae and macroalgae species (*Ulva*, *Enteromorpha*, and *Cladophora*)	European sea bass (*Dicentrarchus labrax* L.) (10 ± 2; initial density, 82 ± 22; final density)	Commercial feed, 44–52% protein (1.5% body weight)	3	3.0 (0.5 ± 0.2 g N m^–2^ day^–1^)- summer (0.09) g N m^–2^ day^–1^) winter	Deviller et al., 2004

**FIGURE 1:**
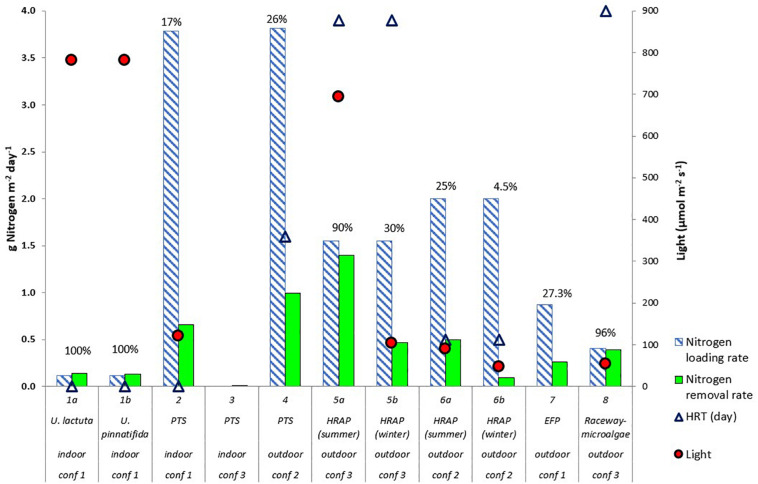
Nitrogen removal and loading rate (g Nitrogen m^– 2^ day^– 1^) by algal reactors in recirculating aquaculture systems (RAS).% value refers to the percentage of nitrogen removed. Maximum photosynthetic active radiation (light – μmol m^– 2^ s^– 1^) used in the studies are shown by red circles. Hydraulic retention time (HRT) (days) are shown by triangles (primary axis). For Study 3, HRT was not given, for Study 7, HRT was 60 days (not included in figure) The RAS configurations of Studies 1–8 are shown in Figure 4. Indoor and outdoor labels in the figure indicate the location of the algae reactors. The algae reactor in Study 1 used two algal species (Cahill et al., 2010), *Ulva lactuca* and *Ulva pinnatifida*. Study 2 (Valeta and Verdegem, 2015), Study 3 (Huang et al., 2013), and Study 4 (SustainAqua, 2009) used a periphyton turf scrubber (PTS). Study 5 (Pagand et al., 2000) and Study 6 (Deviller et al., 2004) used high-rate macroalgal based algal ponds (HRAP), and Study 7 (Gál et al., 2003) used an extensive fish pond (EFP). Study 8 (Li et al., 2019) used outdoor microalgal based raceway as methods to integrate algae in the RAS.

**FIGURE 2:**
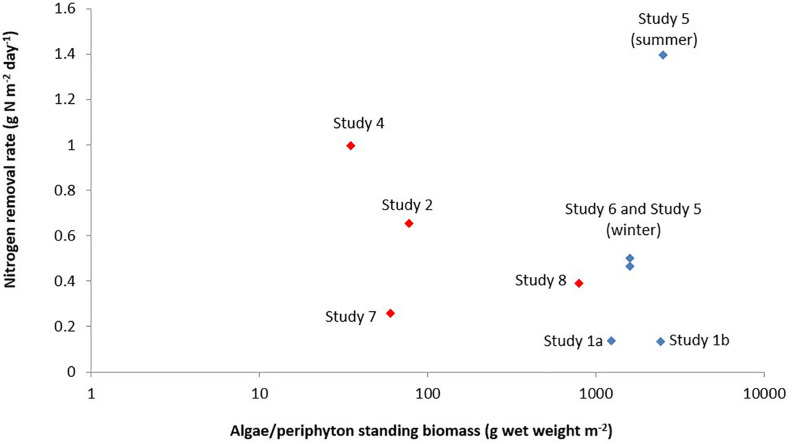
Nitrogen removal rate versus algae/periphyton standing biomass in a recirculating aquaculture system. The red diamonds represent microalgae biomass for the studies of Gál et al. (2003) (Study 7) and Li et al. (2019) (Study 8), and periphyton biomass for the studies of SustainAqua (2009) (Study 4), and Valeta and Verdegem (2015) (Study 2). The periphyton biomass consisted of microorganisms such as phytoplankton, bacteria, fungi, protozoa, and range of invertebrates and detritus. The blue diamonds represent the macroalgae biomass from the studies of Cahill et al. (2010) (Study 1), Deviller et al. (2004) (Study 6), and Pagand et al. (2000) (Study 5). Information on algae for Study 3 was not part of the design of that study, thus, was not provided by those authors.

**FIGURE 3:**
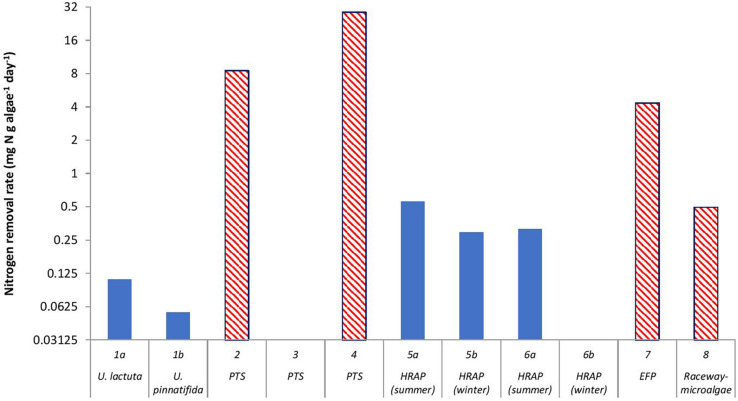
Nitrogen removal rate by algae (mg N g algae dry weight^–1^ day^–1^) in a recirculating aquaculture system. The algal reactor in Study 1 (Cahill et al., 2010) used two algal species, *Ulva lactuca* and *Ulva pinnatifida*. Study 2 (Valeta and Verdegem, 2015), Study 3 (Huang et al., 2013), and Study 4 (SustainAqua, 2009) used a periphyton turf scrubber (PTS), Study 5 (Pagand et al., 2000), and Study 6 (Deviller et al., 2004) used high-rate macroalgal based algal ponds (HRAP), Study 7 (Gál et al., 2003) used an extensive fish pond (EFP), and Study 8 (Li et al., 2019) used outdoor microalgal based raceway as methods to integrate algae in the RAS. The red bars with a diagonal pattern represent microalgae and the blue bars represent macroalgae. Information on algae for Studies 3 and 6b were not part of the design of those studies, thus, were not provided by those authors.

**FIGURE 4:**
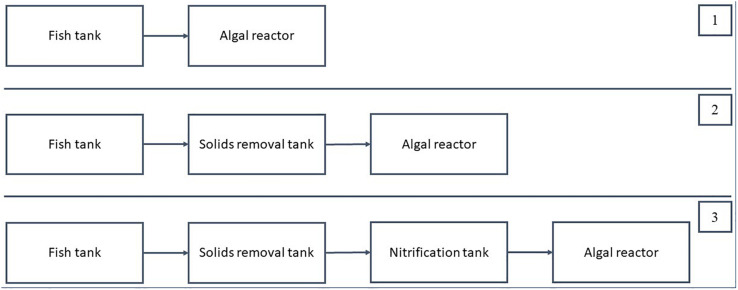
Recirculating aquaculture system (RAS) configurations with algal reactors. After the algal reactors, the water was either fully recirculated back into the RAS, or partly discharged to the environment. Configuration 1 was used by Studies 1 (Cahill et al., 2010), 2 (Valeta and Verdegem, 2015), and 7 (Gál et al., 2003). Configuration 2 was used by Studies 4 (SustainAqua, 2009), 5 (Pagand et al., 2000), and 6 (Deviller et al., 2004). Configuration 3 was used by Studies 3 (Huang et al., 2013) and 8 (Li et al., 2019).

The system configurations are detailed in Supplementary Table 1. (Studies 1–8 refer to labels used in the main text, Figures 1–4 and Supplementary Table 1). ^1^When assuming depth of algae tank is 0.3 m (also for other information which is labeled with an *).

The different RASs that have been integrated with an algae unit (Table 1) could be classified according to the type of algae cultured (macroalgae or microalgae), the location of algae unit (indoor or outdoor), and the different configurations of the RAS. Different fish or invertebrate species, stocking densities, and feeds given affect the nutrients availability in the respective systems. These nutrients are partially digested by the cultivated organisms and became the nutrient source for the algae. In this review, only nitrogen is considered. The nitrogen-loading rate indicates the amount of nitrogen entering the algal reactor per area per day (g m^–2^ day^–1^). The nitrogen removal rate indicates the amount of nitrogen removed by the algal reactor (g m^–2^ day^–1^). Before any comparison can be made between the respective studies, it is important to check how nitrogen loading rates and nitrogen removal rates were calculated in each study.

### Estimation of Nitrogen Loading Rate

The nitrogen loading rate is the amount of nitrogen received per unit area of the algae reactor per unit of time (g N m^–2^ day^–1^). The nitrogen loading rates in the eight studies are shown in Figure 1. In this review, Studies 1 through 8 as referred to Figure 1 will be used in the text. Studies 1 (Cahill et al., 2010), 2 (Valeta and Verdegem, 2015), and 4 (SustainAqua, 2009) reported the nitrogen loading rates measured in their studies. However, Studies 5 (Pagand et al., 2000), 6 (Deviller et al., 2004), 7 (Gál et al., 2003), and 8 (Li et al., 2019) did not report the nitrogen loading rate, and therefore, the nitrogen loading rate was estimated by the authors in this review using the nitrogen concentration and flow rate into the algal reactor or using the percentage of nitrogen removal rate in the studies. Similarly, Study 3 did not report nitrogen loading rates. In this case, it was impossible to calculate the nitrogen loading rate since the mussels cultivated under Study 3 were fed live algae, and no information was given concerning the amount of microalgae fed.

### Estimation of Nitrogen Removal Rate

There were three methods used to estimate the nitrogen removal rate in the respective studies. The first method estimated the nitrogen removal rate from algal growth or algal productivity (Brune et al., 2003). The basis for this method is that the rate of algal productivity (photosynthesis) reflects the rate of nitrogen assimilation of algae (g N m^–2^ day^–1^). The nitrogen assimilation by algae reduces the nitrogen concentration in water, and thus is considered also as nitrogen removal. The nitrogen removal rate by algae is normally expressed per unit area considering the light distribution, which is expressed per unit area. During photosynthesis, inorganic carbon in the form of carbon dioxide (CO_2_) or bicarbonate (HCO_3_) is used as the carbon source and nitrogen in the form of ammonium (NH_4_^+^) (Equation 1) or nitrate (NO^–^_3_) (Equation 2) is used as an N source (Stumm and Morgan, 1995; Ebeling et al., 2006):

(1) $$16\,N\,H_{4}+92\,C\,O_{2}+\,92\,H_{2}\,O+14\,H\,C\,O_{3}^{-}+H\,P\,O_{4}\to C_{106}\,H_{263}\,O_{110}\,N_{16}\,P+106\,O_{2}$$

(2) $$16\,N\,O_{3}^{-}\,+124\,C\,O_{2}+\,140\,H_{2}\,O+H\,P\,O_{4}^{2-}\to C_{106}\,H_{263}\,O_{110}\,N_{16}\,P+138\,O_{2}+\,18\,H\,C\,O_{3}^{-}$$

According to Equation 1, 1 g of ammonium nitrogen assimilated by algae produces 15.84 g of algae biomass. Also, in this formula, carbon comprises 35% and nitrogen comprises 6% of the algal biomass; thus, the ratio of carbon to nitrogen (*C*/*N*) of algae biomass is 5.6:1. This formula can be used to estimate nitrogen assimilation when the algal biomass (given as dry weight or as carbon content) in a system is known. There is also another ratio used for carbon content in algae whereby from the measured algae dry solids, 50% is carbon (Chisti, 2007). Meanwhile, a *C*/*N* ratio of algae of 10:1 also is used (Boyd, 1985) as in Study 7 (Gál et al., 2003). The use of a *C*/*N* ratio of 5.6:1 could lead to a higher estimation of nitrogen removal by algae than using a *C*/*N* ratio of 10:1. The nitrogen content of algae also can be directly determined by nitrogen composition analysis of the algae (Study 4) (SustainAqua, 2009).

Where algal productivity is not available, standing algal biomass (g m^–2^ or g L^–1^) is used as a metric of N removal. This method is normally used in combination with the calculation of the nitrogen budget of a system. A disadvantage is that by using the algal standing biomass, the nitrogen removal rate cannot be determined.

The second method used to determine the nitrogen removal was by measuring nitrogen difference between influent and effluent streams of an algal reactor (Pagand et al., 2000). In the third method, the nitrogen removal by an algal reactor was estimated by comparing difference of nitrogen between a system with algae and a system without algae.

The first method was reported in Study 1 (Cahill et al., 2010), Study 4 (SustainAqua, 2009), and Study 7 (Gál et al., 2003). The second method was reported in Studies 2 (Valeta and Verdegem, 2015), 5 (Pagand et al., 2000), and 8 (Li et al., 2019). The third method was reported in Studies 3 (Huang et al., 2013) and 6 (Deviller et al., 2004).

### Nitrogen Removal Rate in Algae Reactors in RAS

Different nitrogen loading and removal rates as the result from different RAS configurations and culture practices is visualized in Figure 1. Based on Figure 1, variables presented with regards to nitrogen removal rate are nitrogen loading rate, light, hydraulic retention time (HRT) of algae reactors, type of algae, algae cultivation system [periphyton turf scrubber (PTS), high-rate algal pond (HRAP), extensive fish pond (EFP), and microalgal based raceway], location of algae reactor (indoor versus outdoor), and RAS configuration (1, 2, and 3). The concept of PTS in the mentioned studies is also termed as an algal turf scrubber (ATS), where microalgae and other types of microorganisms attach on a substrate prepared for the experiments (Adey, 1982, 1992, 1998; Adey and Purgason, 1998). Therefore, the term PTS will be used in this review. The HRAP term is used to describe the specific characteristics of a pond that is shallow, normally at 0.5 m deep, and intensively mixed (Benemann et al., 1977). The other variables were not plotted in Figure 1, either because the values were controlled by the fish requirement rather than algal requirement (e.g., pH) or because no quantitative data were provided in the studies (e.g., species composition and nutrient composition).

The above-mentioned system variables influenced nitrogen removal rates. From Figure 1, study 5a (Pagand et al., 2000) had the highest nitrogen removal by the algae (1.4 g N m^–2^ day^–1^). This removal rate is comparable with removal rates achieved by algae in aerated pond systems (between 0.5 and 1.8 g N m^–2^ day^–1^, considering the *C*/*N* ratio of algae between 5.6:1 and 10:1 as described in section “Estimation of Nitrogen Removal Rate”) (Brune et al., 2003), and in IMTA systems (between 1.3 and 1.5 g N m^–2^ day^–1^) (Abreu et al., 2011; Ben-Ari et al., 2014). Factors that could explain this high nitrogen removal and which were not demonstrated in other studies were the combination of high light intensity (694 μmol m^–2^ s^–1^) and HRT (3.9 days). Study 5b (Pagand et al., 2000) had a lower nitrogen removal rate than 5a because of the low light intensity during winter. For Studies 1 (Cahill et al., 2010) and 8 (Li et al., 2019), even though the nitrogen removal rate was 100%, which might indicate the efficiency of the algae, the nitrogen loading rates were low compared to other studies. For Studies 2 (Valeta and Verdegem, 2015) and 6 (Deviller et al., 2004), either light intensity or HRT was low, which caused the low nitrogen removal rate. Meanwhile, in all studies, especially for outdoor algal ponds, Studies 4 (SustainAqua, 2009), 6 (Deviller et al., 2004), and 7 (Gál et al., 2003), nitrogen removal could be driven by heterotrophic bacteria, possibly due to high carbon, which was released by bacteria and algal decomposition in the system. This is related to the fact that even though nitrogen removal by algae was not 100%, inorganic nitrogen concentration in the RAS was low (Supplementary Table 2). The effects of algae type, algae cultivation system, location, and RAS configuration on nitrogen removal rate could not be quantified, although these factors might be important. Nevertheless, outdoor systems could be associated with high light intensity, which determines the high nitrogen removal rate by the algae.

From these examples, factors that may affect nitrogen removal rate can be categorized under five main factors: (1) algal biological characteristics (algal growth rate, algal species and species composition); (2) RAS configuration; (3) nutrient availability in RAS (nitrogen loading rate, nitrogen species and nutrient waste composition); (4) algal culture system (outdoor versus indoor algae culture), and algal cultivation technique (suspended or attached); and (5) algal reactor conditions (such as HRT, light, carbon dioxide, dissolved oxygen, and pH). These factors are further discussed in Section “Factors Affecting Nitrogen Removal Rates by Algae.”

## Factors Affecting Nitrogen Removal Rates by Algae

### Algae Growth Rate

It is widely accepted that algae growth rate is expected to be correlated with nitrogen removal rate. A high algal biomass can be an indicator of a high growth rate. However, a high biomass does not guarantee a high nitrogen removal rate because other environmental factors such as light and CO_2_ in the culture system may become limiting.

When a comparison is made between macroalgae and microalgae (Figure 2), the macroalgae biomass (g algae m^–2^ algae reactor) was higher than the microalgae/periphyton biomass. Except for Study 8 (Li et al., 2019), a relatively high microalgal biomass was observed. This could be due to the HRAP technique used in that study that enhanced the growth of microalgae (Brune et al., 2003). However, the removal rate of nitrogen per g algae per day (mg N removed g^–1^ algae day^–1^) by a mixture of microalgae and periphyton was higher than that by macroalgae (Figure 3). This was most likely because the periphyton biomass also comprised of microorganisms, which also took up nitrogen (Azim et al., 2005; Asaduzzaman et al., 2008; Levy et al., 2017). Moreover, Hein et al. (1995) reported that the nitrogen uptake kinetics by algae is size-dependent. The report suggested that small-sized algae could provide a higher surface area for the nitrogen uptake rates, which is why microalgae could have a higher uptake rate than macroalgae.

In most algal reactors in a RAS, multi-species algae were observed instead of mono-species (Table 1). Since algae have different tolerance levels for total ammonia and different affinities toward the respective nitrogen species, presence of multiple species can be beneficial to nitrogen removal in a RAS through niche separation by these species in the systems [please see more elaboration in section “Outdoor Versus Indoor Algae Reactor (Light and Temperature)”].

Meanwhile in mono-species cultures, growth factors are more easily controlled. For instance, in Study 1 (Cahill et al., 2010) a single species of alga (*Ulva lactuca* for treatment 1, *and Ulva pinnatifida* for treatment 2), was used in the reactor, and the culture conditions were set according to the species’ requirements. The use of a mono-species culture for a specific function in a RAS, for example for nitrate removal, would be beneficial if the algae perform well under the RAS conditions.

### Nitrogen Loading Rates and Waste Composition

One of the most striking differences between the respective studies is the nitrogen loading rate (Figure 1). Studies 1 (Cahill et al., 2010) and 8 (Li et al., 2019), which had a low loading rates (0.11 and 0.41 g N m^–2^ day^–1^, respectively), had a 100% removal rate. However, other studies, which had nitrogen loading rates above 0.8 g N m^–2^ day^–1^, had nitrogen removal rates of between 17 and 27%, except for two cases that received high light intensity (690 μmol m^–2^ s^–1^) and low light intensity (46 μmol m^–2^ s^–1^), exhibiting 90 and 5% nitrogen removal rates, respectively. Hence, the nitrogen loading rate and nitrogen removal rates vary greatly between systems. Before the effects of nitrogen loading rates are discussed, factors that determine the nitrogen loading rates will be elaborated first. Waste composition is also discussed under this topic because factors that determine the nitrogen loading rate also affect waste composition.

#### Factors Determine Nitrogen Loading Rates and Nutrient Composition of Waste

In a RAS, the nitrogen loading rate tends to be dependent on the types of culture, stocking density, and the RAS configuration. Metabolism, nutrient requirement, and husbandry of fish, crustaceans, and mollusks differ, and therefore different nutrient-loading rates are observed (Butterworth, 2010; Meriac et al., 2014b; Nunes et al., 2014). For example, the indoor RAS in Study 2 (Valeta and Verdegem, 2015) maintained tilapia at densities ranging between 30 and 70 kg m^–3^, producing a nitrogen loading rate into the algae reactor of 3.79 g nitrogen m^–2^ day^–1^. In Studies 5 (Pagand et al., 2000) and 6 (Deviller et al., 2004), indoor RASs contained sea bass and the maximum stocking densities used were 100 and 80 kg m^–3^, respectively. Even though the algal reactors in these studies received only 6–10% input from the fish culture tank, the nitrogen loading was high (Figure 1). However, for Study 4 (SustainAqua, 2009), even though the stocking density in the carp pond was low (15 kg m^–3^), a high nitrogen loading rate was observed in the algal reactor because it received 100% input from fishpond. In these studies, therefore, RAS configuration also played a role in determining the nitrogen loading into algae reactors, which will be discussed in the following section.

##### RAS configuration

Based on the studies of RAS, which included an algal reactor (Table 1), three different RAS configurations can be conceptualized to enhance the effectiveness of ammonia removal (Figure 4). In these configurations, only units supplying input to the algal reactor are considered. The first configuration comprises a fish culture unit and an algal reactor. The second configuration connects three components, a fish culture unit, a solids removal unit, and an algal reactor, and the third configuration is the same as the second except that a nitrification unit is integrated before the algal reactor.

The first RAS configuration uses an algal reactor as the only means to remove nitrogen. Since there is no nitrification unit installed, the algal reactor must be designed for a complete removal of the nitrogen excreted by the fish, as in Studies 1 (Cahill et al., 2010), 2 (Valeta and Verdegem, 2015), and 7 (Gál et al., 2003). The waste composition, that is, carbon to nitrogen (*C*/*N*) ratio of the waste entering the algal reactor, was expected to be high under this setup because particulate waste entered the algal reactor.

In the second configuration, the algal reactors served as a post-solids removal treatment unit since approximately 70–80% of the particulate waste was removed in the solids removal tank, as in Studies 4 (SustainAqua, 2009), 5 (Pagand et al., 2000), and 6 (Deviller et al., 2004). The solids removal process was performed in conventional RAS to support the biofilter, which requires a low *C*/*N* ratio (preferably between 0 and 1) (Zhu and Chen, 2001); therefore, under the second configuration, the algae reactor would receive a low *C*/*N* ratio. With the solids removal process, the *N*/*P* ratio of the waste entering the algae reactor would also be affected because particulate *P* would be removed in the solids removal unit. In this configuration, the amount of water channeled from the solids removal unit can be controlled. For example, in Study 4 (SustainAqua, 2009), 100% of the water was channeled into the algae pond. Meanwhile, for Study 6 (Deviller et al., 2004), only about 10% of the water was channeled into the algae pond.

For the third configuration, an algal reactor is located after the nitrification reactor. The nitrification reactor reduces the ammonia concentration and increases the nitrate concentration, allowing the algae to function specifically for the removal of nitrate-N. As reported in Studies 3 (Huang et al., 2013) and 8 (Li et al., 2019), which used this configuration, the nitrate level is significantly lower in the RAS with algae than in the control RAS without algae. Thus, the second and the third configurations allow the flexibility to control nitrogen loading and size of the algae reactor, including flow rates.

#### Effects of Nitrogen Loading Rate and Waste Composition on Nitrogen Removal Rate

##### Total ammonia tolerance of algae

Nitrogen loading rate determines the concentration of nitrogen in the water and affects algae growth. Generally, an ammonium-N concentration below 1.09 mg L^–1^ would not affect the growth of microalgae (Collos and Harrison, 2014). For the marine phytoplankton species, *Nephroselmis pyriformis*, unionized ammonia-N at 0.0328 mg L^–1^, and ammonium-N at 3.14 mg L^–1^ were found to be toxic (Källqvist and Svenson, 2003). Meanwhile, Collos and Harrison (2014) reviewed 45 freshwater and 68 marine microalgae species and concluded that optimum and toxic levels of ammonium differ between microalgae species (Table 2). In these studies, unionized ammonia toxicity was observed mainly when the pH was >9, and ammonium toxicity occurred when the pH was <8. Collos and Harrison (2014) suggested *Nannochloropsis* sp. as a suitable candidate for aquaculture systems, since this species can tolerate total ammonia levels of 12 mg L^–1^ at pH 8 (Hii et al., 2011). Further, *Chlorella vulgaris*, which is a common species in aquaculture ponds, was reported to tolerate an ammonia-N concentration of 280 mg L^–1^ at pH 7.0 (Tam and Wong, 1996).

**TABLE 2:** Toxic and optimum concentration of ammonium for different microalgae species [Collos and Harrison (2014) and references therein].

	Toxic	Optimum
		
	Ammonium level (mg L^–1^)	Ammonium level (mg L^–1^)
Chlorophyceae	702.4	136.9
Cyanophyceae	234.1	45.0
Prymnesiophyceae	41.4	25.2
Diatomophyceae	64.8	6.1
Raphidophyceae	45.0	4.7
Dinophyceae	21.6	1.8

##### Preference of nitrogen species

The preference of algae for the reduced forms of nitrogen (ammonium, urea, dissolved free amino acids and adenine) or the oxidized form of nitrogen (nitrate) could affect the nitrogen removal rate by algae (Dortch, 1990; Yuan et al., 2012). Most algae prefer ammonium as the nitrogen source because less energy is needed compared to other forms of inorganic nitrogen, such as nitrate (Dortch, 1990; Hii et al., 2011). Only when ammonium was not detected was nitrate uptake positive, and correlated with phytoplankton cell size (Yuan et al., 2012). The common view of the nitrogen cycle is that bacteria decompose organic nitrogen and that algae use inorganic nitrogen. There is some overlap, as both bacteria and algae use both organic and inorganic nitrogen (Kirchman, 1994; Allen et al., 2002; Bronk et al., 2006). When inorganic nitrogen is limited, algae can use urea as a nitrogen source (Bradley et al., 2010). For example, *Prochlococcus* spp. was found to assimilate organic nitrogen in a low-nutrient environment (Zubkov et al., 2003). Yuan et al. (2012) found that after ammonium is depleted, algae would use organic N (including urea and amino acids) rather than nitrate. *Nannochloropsis oculata* and *Stigeoclonium nanum* prefer ammonium to nitrate, in contrast to *Chlorella vulgaris* that prefers nitrate (Podevin et al., 2015; Ramli et al., 2017).

While most green algae and cyanobacteria prefer ammonium to nitrate, diatoms, and dinoflagellates prefer nitrate over ammonium (Dortch, 1990; Domingues et al., 2011). In the Gulf of Riga, Latvia, only diatoms were able to use the oxidized form of nitrogen (nitrate), while other phytoplankton such as cryptophytes, dinoflagellates, and filamentous cyanobacteria were able to use reduced forms of nitrogen (Berg et al., 2003). There is mounting evidence that supports this finding (Glibert et al., 2014a,b, and references therein). For instance, the occurrence of harmful algal blooms was encouraged under an elevated *N*/*P* condition with a high concentration of ammonium or urea. This finding has led many researchers to recommend strategies that the effluent entering the San Francisco Bay Delta in California should have a high nitrate concentration through nitrification in order to encourage diatoms, which are more beneficial for fish and higher trophic level consumers (Glibert et al., 2014a,b). In a RAS, where the nitrate concentration can become too high, the use of diatoms to remove nitrate should be encouraged (Ramli, 2018). Diatoms are commonly found in aquaculture ponds (Yusoff et al., 2002; Khatoon et al., 2007; Shaari et al., 2011) and HRAP connected to RAS (Pagand et al., 2000). This proves that aquaculture waste is suitable for diatom culture. Nonetheless, Khatoon et al. (2007) observed that even though diatoms dominated the periphyton community at the beginning, after the 21 days of experiment, biofouling activities started to occur and dominated the substrate after 60 days of the experiment. However, for indoor RAS, no study on the use of substrate, except for Huang et al. (2013) and Valeta and Verdegem (2015), and no biofouling activities on algae substrate were reported. Besides biofouling, another important consideration for diatom culture is silicate concentration. Normally, in diatom cultures, silicate is added to support cell wall development (Bondoc et al., 2016). Therefore, experiments aiming to develop guidelines for silicate requirements to integrate diatom culture in RAS are needed.

##### Effects of waste composition (C/N and N/P ratio)

Nitrogenous waste composition may influence the nitrogen assimilation (nitrogen removal) by the algae (Glibert, 2012). In aquaculture systems, waste composition influences the contributions of heterotrophic, autotrophic, or phototrophic processes to waste removal (Avnimelech, 1999; Ebeling et al., 2006). A carbon to nitrogen (*C*/*N*) ratio of more than 10 will encourage heterotrophic processes, while a *C*/*N* ratio between 6 and 7 will encourage photosynthetic process by microalgae (Ebeling et al., 2006). When decomposition of microalgae is high, the *C*/*N* ratio in the water will increase, which favors heterotrophic processes. Sometimes, even though high microalgal abundance is observed, heterotrophic processes may dominate the removal of nitrogen, which has been observed in an intensive tank system receiving a high feed load (Rakocy et al., 2004).

The nitrogen to phosphorus ratio (*N*/*P*) affects the algal community composition (Heisler et al., 2008; Glibert, 2012). In turn, algal composition affects the nitrogen removal in an ecosystem. For example, in a community where cyanobacteria dominate then ammonium removal is high, whereas in a community where diatoms dominate then nitrate removal is high (Glibert et al., 2014b). A specific example of the *N*/*P* ratio affecting algal growth was reported for *Tisochrysis lutea* and *Nannochloropsis oculata*; a *N*/*P* ratio of 20 improved their growth while a *N*/*P* ratio of 120 reduced their growth (Rasdi and Qin, 2015). Increased or reduced algal growth under a certain nutrient composition will have a direct effect on the algal composition. In addition to the waste composition (*N*/*P* ratio), nutrient concentrations, especially of nitrogen, phosphorus and silica, influence the microalgal community structure in ponds (Yusoff and McNabb, 1989, 1997). For instance, Liu et al. (2011) showed that the effect of the *N*/*P* ratio is dependent on the nutrient concentration for *Microcystis aeruginosa*. When the initial nitrogen concentration was 10 mg L^–1^, an *N*/*P* ratio of 16 was the optimum for their growth, but when the initial *P* was 1 mg L^–1^, a *N*/*P* ratio of 40 was found to be optimum (Liu et al., 2011).

In aquaculture ponds, the microalgal community composition is highly dynamic; thus, an algae reactor connected to an aquaculture pond should experience similar dynamics. Shaari et al. (2011) reported that before shrimp were introduced into a culture pond, cyanobacteria dominated. After the shrimp had been introduced, diatoms dominated. In contrast, Yusoff et al. (2002) found that diatoms were dominant at the early and middle stages of shrimp culture: toward the end of the culture period, cyanobacteria were dominant. The result was supported by the study of Casé et al. (2008) who found that diatoms were replaced by cyanobacteria toward the end of the shrimp culture.

### Outdoor Versus Indoor Algae Reactor (Light and Temperature)

From the RAS studies, the major practical differences between outdoor and indoor algae culture are the options to control light and temperature. Light is an important parameter affecting algal growth and influencing nitrogen assimilation by the algae. The saturation irradiance observed for many algae species was between 100 and 400 μmol photons per m^2^ per second (Necchi, 2004). However, light availability in the water is limited by water turbidity; therefore, even though enough light might be provided, the light availability for algae might be restricted (Anthony et al., 2004; Tait et al., 2014).

During summer when light irradiance is high, an outdoor culture system that received sunlight had a higher nitrogen removal rate than an indoor algae reactor (Figure 1). On the other hand, the outdoor algae cultures are exposed to fluctuations in sunlight irradiance due to the day/night cycles and changes in weather conditions and seasons. A wide range of irradiance was reported, between 46 and 1700 μmol photons per m^2^ per second (Figure 1 and Table 1). Quick changes in irradiance pose a high risk of culture collapse (Brune et al., 2003; Blanken et al., 2013).

Valeta and Verdegem (2015) (Study 2) applied artificial light with an intensity of 120 μmol photons per m^2^ per second. When artificial light is supplied, no fluctuation of light intensity occurs. Microalgae can use all the photons in the photosynthetic active radiation (PAR), which have a wavelength between 400 and 700 nm (Blanken et al., 2013). However, red light (660 nm) is the optimum light for photosynthesis (Chen et al., 2010; Cuaresma et al., 2011). Therefore, by using artificial light, for example LED light, the specific wavelength required can be supplied (Schulze et al., 2014). However, it is well accepted that the costs of artificial light for culturing algae is high. Blanken et al. (2013) reported that the cost of artificial light is $25.3 per kg dry-weight biomass (at the time when the paper was published, 1.34 US dollars was equal to 1 €). From the point of biofuel production, this value would make the cost of algae production 25 times more expensive than using sunlight. Profitable biofuel production requires a cost under $1.3 per kg dry-weight biomass (Wijffels et al., 2010; Slade and Bauen, 2013). Therefore, the lighting cost could be an issue and impede integration of algae into a RAS.

Temperature is important because it influences the rates of enzymatic reactions that occur during photosynthesis. With a 10°C temperature increase, the enzymatic reactions are doubled (Goldman and Carpenter, 1974), thus doubling the nutrient uptake by the algae. In an outdoor culture where temperature cannot be controlled, minimizing the temperature fluctuation is a challenge, especially in areas that experience drastic temperature fluctuations (Supplementary Table 1). During winter, the water temperature can be sufficiently low that it results in a low nitrogen removal rate, as observed in Pagand et al. (2000) and Deviller et al. (2004) (Studies 5 and 6). Again, the advantage of an indoor reactor is that temperature can be controlled, enabling stable nitrogen removal all year round.

### Effects of the Algae Cultivation Method

There are two algal culture methods that might be used in a RAS; namely, suspended or attached. From the comparison given (Figure 1), the method of cultivation did not seem to influence the nitrogen removal rate because of the interacting effects of other factors such as light and CO_2_. Nonetheless, each method requires specific management, for example, reactor preparation or mixing, which have a direct impact on algae growth, and thus the nitrogen removal rate by the algae. For suspended culture, the preparation of the reactor is relatively simple, with a simple pond or a tank as enough. The HRAP is an intensive waste water treatment pond, which combines wastewater treatment, reclamation, and algal biomass production (Benemann et al., 1977). The pond is shallow and continuously aerated normally by paddle wheel to expose the algal cells to sunlight, to create a homogenous chemical environment and to avoid pond stratification (Brune et al., 2003). Meanwhile, in a raceway, algae are kept in suspension by a paddle wheel (Vonshak and Richmond, 1988). Reports on the use of suspended algae in an indoor algae reactor were not found.

The attached culture method refers to an ATS or PTS, which use substrates to support the growth of algae mats (Adey et al., 1993, 1996, 2013; Azim et al., 2005). In ponds, vertical poles, for example bamboo, fixed at the bottom often are used as a substrate (Azim et al., 2002; Richard et al., 2010). The substrates provide additional surface area for algae growth (Rahman et al., 2008; Asaduzzaman et al., 2010a,b). Air lifts or paddle wheels also are used to keep the water column mixed. The pond depth is also shallow, ranging between 0.5 and 1.0 m. This is unlike the suspension pond, where microalgae can have equal exposure to light through proper mixing. The bottom section of the water column receives less light than that close to the surface. Even so, the presence of substrate contributes to a large portion of autotrophic productivity by the periphyton community. Guiral et al. (1993) reported that a pond with a periphyton community realized 7.9 g C m^–2^ day^–1^ productivity, where this value was 4.5 times higher than in a pond with a phytoplankton community. Also reported in Azim et al. (2002), periphyton is accounted for 50% of the total primary productivity in a fishpond. In an indoor RAS, a flat wire mesh can be used as substrate and laid horizontally to provide an optimum surface area for the algae mats (Valeta and Verdegem, 2015) (Study 2). For mixing, a tipping bucket, which is located at the top section of the substrate, is filled and emptied continuously to create waves over the substrate in order to move nutrients across the substrate and facilitate gas exchange. In Huang et al. (2013), the ATS (60 cm long × 60 cm wide; mesh size = 1.3 cm × 1.3 cm) was made from plastic mesh. Water was pumped from the RAS over a vertically positioned ATS and returned through a sump to the RAS. This setup allowed light to easily illuminate the biofilm, promoted CO_2_ and O_2_ exchange, and facilitated homogenous distribution of nutrients passing through the ATS.

The major disadvantage for these two methods is that to capture enough light to control the ammonia, a larger surface area is needed. However, in many RAS, the surface area is of great concern. Since the surface area problem is not limited to the application of algae in a RAS, a recent innovation was made for the algae cultivation method where a solid-state biofilm method was applied (Naumann et al., 2013). The basic principle of this method is that algae were cultivated on vertically orientated twin-layer modules, which consisted of two ultrathin layers. The first layer is a macroporous layer where the algae culture medium passes through by the force of gravity, and the second layer is a microporous layer where the microalgae biofilm is attached (Naumann et al., 2013). The vertical arrangement of the biofilm substrates allows more efficient use of the surface area and exposure to light (Cuaresma et al., 2011). Blanken et al. (2014) used a very similar approach by applying a microalgal biofilm on a rotating Algadisk, which was vertically positioned and placed in a container. The disk rotated between the air (light) and water (dark) phase, and nutrients were supplied to the microalgal biofilm during the latter phase. When *Chlorella sorokiniana* was cultured using the Algadisk method, an algal productivity of 20.1 ± 0.7 g per m^2^ disk surface per day was observed. This productivity would be equal to the removal of 1 g N m^–2^ day^–1^ [using the estimation method used in Gál et al. (2003)] (Study 7), which was higher than the nitrogen removal reported by Valeta and Verdegem (2015), (Study 2) of 0.66 g N m^–2^ day^–1^ when using an indoor PTS. The Algadisk concept may be better than the PTS because of the optimum use of the surface area.

### Effects of CO_2_, O_2_, and pH

Unlike light and temperature, the pH, carbon dioxide, and oxygen values are directly affected by the rate of photosynthesis and respiration in a RAS. In a RAS, the pH could become the least of the problems for algae, because the pH in a RAS is kept close to neutral for fish culture. Normally in a RAS relying on nitrification, the pH is kept above 6 by supplying bicarbonate to compensate for the loss in alkalinity due to nitrification. As shown in Supplementary Table 1, in all studies the pH was maintained between 6.5 and 8.4. By employing photosynthesis in a RAS, the annual amount of bicarbonate addition was reduced, despite low light irradiance during winter as in Study 6. In Study 5, the treated water had a higher pH level than the untreated water. The measurement was taken at midday when photosynthesis was at the highest rate. However, no pH value was reported during dark hours; therefore, the effect of pH on the algae during dark hours was unknown. Nonetheless, for a RAS set-up, the fish tank is separated from the algae tank, and the pH in the fish tank is controlled. Therefore, the fluctuation of pH in the algae reactor has minimal effect on the fish.

A RAS is a highly aerated system to supply enough oxygen for fish and bacterial respiration. Saturation higher than 100% is applied at the water inlet to prevent oxygen depletion (Bregnballe, 2015). In outdoor aquaculture systems such as ponds, diel oxygen fluctuations caused by photosynthesis are reported. Meanwhile, in an algal reactor, oxygen produced by algae could create super-saturation, which could negatively affect algae growth (Chisti, 2007). The oxygen is removed from an algal reactor by degassing through proper mixing. The different dissolved oxygen requirements of a RAS and an algal reactor should be taken into account when including an algae reactor in a RAS.

In addition, due to the highly aerated environment in a RAS, CO_2_ insufficiency can become a serious problem for algae. Fish require oxygen, which can be produced by the algae, and in return, the CO_2_ produced by fish respiration can be absorbed by the algae. How possible interactions between CO_2_ and O_2_ concentrations in RAS affect fish and algae production in RAS is insufficiently explored. The requirements of CO_2_ by the algae and of oxygen by the fish should be a complementary process when algae are integrated in a RAS. The mass transfer of O_2_ and CO_2_ should be monitored to provide solid proof for the supposed mutual benefit and to develop management criteria, which guarantee optimization of this synergistic effect. Nonetheless, until any solution for the synergistic effects can be achieved by algae and fish, the CO_2_ insufficiency in algal tank can be avoided by supplying pure CO_2_ gas as normally practiced in commercial algal photobioreactors.

### Effects of Hydraulic Retention Time

Generally, flow rates through fish tanks in a RAS are set to supply enough O_2_ for the fish. Flow rates are also important to guarantee that solid and dissolved wastes (CO_2_, total ammonia, dissolved organic carbon) are quickly transferred out of the culture tanks. This means that in general, short HRT prevail in the fish tanks of a RAS. For a culture tank less than 1 m^3^, an HRT of 10 min is quite normal, but for culture tanks of more than 1 m^3^, an HRT of 30 min or more is needed (Timmons and Ebeling, 2007). In addition, the type of the solid removal system used in a RAS sets different requirements for the HRT for proper solid waste removal. Normally the longest HRT applied in solid waste removal systems or settling basins is 15–30 min (Liao and Mayo, 1974). Further, fluidized bed sand biofilters that use fine sand particles require a longer HRT than other bio-filtration systems. However, fluidized bed sand biofilters are not commonly used because most RAS are operated under a short HRT in the culture tanks. In contrast, algae reactors require a longer HRT for the algae to grow.

The HRT of an algae reactor influences nutrient, CO_2_, and O_2_ transfer and therefore affects the algal growth rate (Inoue and Uchida, 2016). The applied HRT in the algal reactor will affect the gradients of nutrients, pH, CO_2_, and O_2_ along the reactor. An HRT that is too short will not ensure complete nutrient removal by the algae, whereas an HRT that is too long may cause starvation of the algal cells (Larsdotter, 2006b; Anbalagan et al., 2016). The HRT of an algae reactor should not exceed the time required to maintain the growth rates of algae in the photobioreactor (Larsdotter, 2006b). An HRT less than 0.5 days causes a washout of algae cells and a HRT of 2–3 days is recommended to obtain maximum biomass yield at 12–25°C and 190–450 μmol m^–2^ s^–1^ (Takabe et al., 2016). However, a relatively short HRT is normally used in algal reactors, which might explain the low nitrogen removal rates achieved (Supplementary Table 1). The HRT for the algal reactor will determine the size of the reactor. The longer the HRT, the larger the algal reactor required. Nonetheless, even for a short HRT, the size of the algae reactors used were one to two times the size of the fish culture vessel (Supplementary Table 1). The size of the algae reactor is expected to be one of the main factors influencing the farmers’ choice of which type of algal reactor to install in their RAS.

## Cost-Benefit Analysis of RAS-Photobioreactor Integration

Culturing microalgae using aquaculture wastewater has been found to be efficient (Yusoff et al., 2001; Guo et al., 2013). In this way, the cost of nutrients and water for the algae can be eliminated. It was reported that the cost to produce microalgae using wastewater from a fish farm in a tubular photo bioreactor (PBR) was 36€ kg^–1^ dry weight (Michels, 2015) (for this estimation, microalgae were cultured in a tubular PBR with a total area of 1,000 m^2^. Sunlight and a low-cost temperature controller were used. The average microalgae productivity was 0.3 g L^–1^ day^–1^ at an average biomass concentration of 0.7 g L^–1^ and PAR at 11.8 mole m^–2^ day^–1^). Meanwhile, Oostlander et al. (2020) estimated a cost of €43- kg^–1^ cost production of microalgae in an aquaculture hatchery, which uses tubular reactors having a total areas of 1,500 m^2^. In this review, estimation of cost by Michels (2015) is used for the following analysis.

Current interest concerns how integration of microalgae in a RAS could affect the RAS total production cost. From Timmons et al. (2002), the cost of producing tilapia was 2.06 € kg^–1^ (1.76 $US kg^–1^, 1 € = 1.17 US$). The tilapia were produced in a RAS facility producing 590,000 kg tilapia per year. The stocking density applied was 100 kg m^–3^. It was assumed that the tilapia were fed at 2.5% body weight per day, with feed containing 32% crude protein. Therefore, for 100 kg m^–3^ production, 2.5 kg feed would be given per day. This would produce 62 g ammonia-N day^–1^, using the same assumptions as in Section “INTRODUCTION.” Considering that the nitrogen content in microalgae dry matter is 6% (Equation 1, see section “Integrating RAS With Algal Reactor”), then 1,033 g microalgae biomass is required to take up 62 g ammonia-N per day. For simplification, 1,000 g (1 kg) microalgae dry weight is taken as the final value.

At a production cost of 2.06 € kg^–1^, 206 € is needed to produce 100 kg tilapia. One kg microalgae is needed to assimilate all the ammonia-N, and the cost of microalgae production was 36€ kg^–1^ dry weight. Therefore, the cost addition by microalgae is about 17.5% of the cost for producing tilapia. However, if artificial light is used, the algal production cost increases by 23€ kg^–1^, raising the cost of tilapia production in the RAS by 29% (see section “Factors Affecting Nitrogen Removal Rates by Algae” on light).

From the aspect of water use, based on a productivity of 0.3 g L^–1^ day^–1^ achieved by Michels (2015), then 1,000 g algae dry weight would require 3,333 L (3.3 m^3^) of photo bio-reactor. Therefore, 3.3 m^3^ of microalgae culture capacity is needed to remove the ammonia-N produced by a 1 m^3^ culture tank in a RAS.

From Ngoc et al. (2016), the cost of producing *Pangasius* was 97.92 € per 100 kg of fish. In this study, 608 tons of *Pangasius* ha^–1^ year^–1^ was produced in a large-scale (>3 hectares) *Pangasius* pond-RAS. Integrating microalgae production would increase the cost for producing *Pangasius* by 37%. Therefore, in the setup used by Ngoc et al. (2016), PBR might not be a suitable method and hence, an outdoor HRAP or periphyton pond would be a more suitable alternative for reducing the cost for algae integration in the RAS.

Even though addition of an algal reactor into RAS could add to the production cost, it is important to note that some countries charges levy for every unit of pollution discharged to the environment. For example, in the Netherlands, the levies that are charged to farmers are based on unit of pollution (p.u.), which refers to oxygen-consuming substances discharged per year (Herman et al., 2017). One unit of pollution is equals to 49.6 kg O_2_ per year, and the charge is €32/p.u. (Warmer and van Dokkum, 2002). Based on the prior estimation that 1033 g of microalgae are needed to assimilate 62 g ammonia-N per day ammonia produce in a one cubic meter of fish tank, the photosynthesis that will use 62 g ammonia-N per day will produce 940 g oxygen per day (based on Equation 1). Therefore, for 1 year, it can be estimated that 343.1 kg oxygen will be produced by the microalgae. This oxygen is equals to 6.9 p.u., which value at €221 per year.

The advantageous effect of algae integration on cost is also dependent on the value of the algae. For example, the market value of common microalgal species such as *Chlorella* and *Arthrospira* (formerly known as *Spirulina*) biomass are 44 and 42 USD kg^–1^, respectively (Barkia et al., 2019). These values are higher than the algal production cost estimated above, and if the biomass can be produced in a RAS, it will increase the total revenue of the RAS. In terms of water volume, adding three times the volume of the fish culture tanks to culture algae in a RAS raises system and production costs. Therefore, the percentage of nitrogen immobilized in the algal biomass might be reduced to the level that is economically acceptable. Nonetheless, technological advancement in algae cultivation is moving toward higher algal productivity and lower cost. The same development is also occurring in a RAS. If cost reductions can be realized in algal systems and in RAS systems, then cost-effective integration of an algal reactor in a RAS might become feasible.

## Conclusion

This review identifies two challenges related to algal integration in RAS: first, the practical feasibility for improving nitrogen removal performance by an algal reactor in RAS, and second, the economic feasibility of integrating an algal reactor in RAS. This review demonstrates that algae could be used to remove inorganic nitrogen in RAS. The maximum removal that was achieved by one of the RASs reviewed was 1.4 g N m^–2^ day^–1^. The main factors that determine the high removal rates are light and HRT. Besides these factors, RAS configuration is important for the effectiveness of the algal reactor. RAS configuration relates directly to the HRT that can be applied for the algal reactor. When an algal reactor receives a fraction of wastewater and is placed after the solids waste removal unit or nitrification unit, this configuration could allow flexibility to determine the HRT of the algal reactor. Since the performance of nitrogen removal rates by algae is determined by HRT, this will affect the size (area or volume) of the algal reactor due to the time required for nutrient uptake by algae and large surface area needed to capture enough light. Another important factor that determines nitrogen removal rate is the nitrogen loading rate. From the review, the maximum nitrogen loading rate that allows the maximum nitrogen removal rate by algal reactor remains unknown. Therefore, the importance of these three factors (light, HRT, and nitrogen loading rate) for maximum nitrogen removal by the algal reactor in RAS warrants further investigation. Future work also should focus on implementing a practical and easy-to-manage algal reactor for RAS. The growth and efficiency of the algae in assimilating inorganic nitrogenous waste in RAS must be one of the top design priorities.

Regarding economic feasibility, current algae management and cost structure might hinder the integration of algae in a RAS. However, we believe that future technological advancements in algal cultivation methods (such as improved algal reactor designs and low-cost artificial light) will make algae integration more economically feasible. Advancement in algal cultivation technology will continue, as algae is a high-value material, which are highly sought by the pharmaceutical, animal feed, and energy industries. Limited availability of natural resources such as land and water, as well as possible water discharge regulations will also be driving factors to integrate algae in fish farming activities including RAS.

Our work also can be used as a guideline for choosing many different available methods to ensure a high removal rate for nitrogen by integrating algae in the RAS. The authors suggest the following step-by step process for the integration of algae in a RAS:

(1) The role of the algae in a RAS should be specified, either to utilize total ammonia, or nitrate because this would determine the location of algal tank in the RAS, which is related to RAS configuration. For ammonia removal, the optimal location to integrate the algal reactor can be between the solids removal and biofiltration units where the ammonia concentration is high and the dissolved oxygen level is low. For nitrate removal, location of the algal reactor can be between the bio-filtration unit and the sump, where the nitrate concentration is high.
(2) Algae selection should be done according to their functions in the system, either to remove ammonia or nitrate only, or to be harvested as live feed or for other commercial purposes. A single-species or multiple species of algae can be integrated in the RAS for ammonia and nitrate removal, and for commercial purposes. For ammonia or nitrate removal only, a natural mixed population of algae is also an option.
(3) Algae cultivation technique (suspended or attached) can be chosen based on the algae selected; for example, the suspended technique is more suitable for planktonic algae while attached technique is more suitable for benthic algae.
(4) The operational conditions of an algal reactor, such as HRT, mixing rate, and light, should be optimized for algal growth.
(5) The algal reactor could be integrated as part of the RAS configuration or at the end of the RAS before the water is discharged to the environment. The benefit of having an algal reactor as a within-RAS component is that algae will increase the purification degree of RAS, thus decreasing the water exchange rate. Meanwhile, integrating an algal reactor as an end-of-pipe waste treatment could be easy and efficient to reduce waste discharge from RAS. This could reduce waste discharge fees where they are applied.

## Author Contributions

NR, MV, and JV conceived of the presented idea. NR wrote the manuscript with the help of MV, JV, FY, KN, and NN. All authors contributed to the article and approved the submitted version.

## Conflict of Interest

NN was employed by company Bluescientific Shinkamigoto Co. Ltd. The remaining authors declare that the research was conducted in the absence of any commercial or financial relationships that could be construed as a potential conflict of interest.
