# Supplementary material for: Integration of Algae to Improve Nitrogenous Waste Management in Recirculating Aquaculture Systems: A Review
Source: Front Bioeng Biotechnol. 2020 Sep 4;8:1004. doi: 10.3389/fbioe.2020.01004 (PMC7498764; doi:10.3389/fbioe.2020.01004)
Supplement: Supplementary file 1 [file Table_1.pdf]

Supporting information

Supp. Table 1 System configurations of recirculating aquaculture systems (RAS) integrated with algae. The rates of nitrogen removal of algal reactors are shown in Table 1. (Studies 1 – 8 refer to labels used in the main text, Figures 1, 2, 3 and 4 and Supp. Table 1).

**References**	**RAS configurations**	**System flow rate, L min^{-1}**	**Plant tank flow rate, L min^{-1}**	**Hydraulic retention time, HRT, (day)**	**Algae tank surface area, m^2 (volume, m^3)**	**Algae reactor: fish tank**	**Study duration, days**	**T, °C**	**pH**	**Animal tank's dissolved oxygen, mg L^{-1}**	**Light intensity for plant (photoperiod, L:D)** µmol m^{-2} s^{-1}
**Microalgae**											
**<u>Study 2</u>** RAS - Periphyton turf scrubber, (PTS) (Valeta and Verdegem, 2015)	Fish tank - PTS - sump	6.7	6.7	0.002	2 m^2 (0.02 m^3)	n.a	49	27 to 28	7.6±0.3	6.15±0.52	120.68 (18:6)
**<u>Study 3</u>** RAS – PTS (Huang et al., 2013)	Mussel trough-sump/biofilter - PTS-	23.3	n.a	n.a	0.36 m^2 (n.a)	n.a	91	22.1±1.6	8.72± 0.3	8.4±1.6	Day- natural light (no intensity mentioned) Night- 1034
**<u>Study 4</u>** RAS – PTS pond (SustainAqua, 2009)	Fishpond-sedimentation pond – periphyton pond	900	434	1.6	1000 m^2 (1000 m^3)	3:1	180	n.a	n.a	n.a	n.a
**<u>Study 7</u>** Combined Intensive-extensive	Outdoor pond system	23148		60	200000 m^2 (200000 m^3)	13.5:1	1095	n.a	n.a	n.a	n.a

RAS (Gál et al., 2003)											
**Study 8**<br>RAS-raceway (Li et al., 2019)	3 fish tanks - particle separator - mechanical filter – biofilter - algae HRAP	67	3	4	12	6:12	60	19.4 ± 2.3	7.2 ± 0.2	8.6 ± 2.6	54
**Macroalgae**											
**Study 1a**<br>*Ulva lactuca*<br>(Cahill et al., 2010)	Fish tank - alga tank	10	10	(0.003)	(0.04 m^3)	1:1	14	9.04±0.22	8.4	n.a	207 to 782
**Study 1b**<br>*Ulva pinnatifida*<br>(Cahill et al., 2010)	Fish tank - algal tank	10	10	(0.003)	(0.04 m^3)	1:1	14	9.04±0.22	8.4	n.a	207 to 782
**Study 5**<br>RAS - outdoor high rate algal pond, (HRAP) (Pagand et al., 2000)	2 fishponds - particle separator - mechanical filter - algae HRAP	500	1.04	(3.9)	11.8 m^2 (5.9 m^3)	1:1.7	540	24 (summer)<br>7 (winter)	7.6 (midda y:9)	10 to 15 (midday)<br>>5 (morning)	104 - winter<br>694 - summer
**Study 6**<br>RAS - HRAP (Deviller et al., 2004a)	2 fishponds - particle separator- mechanical filter - algae HRAP	167	11.7 to 16.7	(0.5)	24 m^2<br>(12 m^3)	3:1	365	21 to 26<br>(summer)<br>12 (winter)	6.9 to 7.8	6 to 9	46 – winter<br>89 - summer

Supp. Table 2 Aerial algal biomass (g wet weight m^{-2}) or volumetric algal biomass (g wet weight m^{-3}) (given in parenthesis) and total ammonia nitrogen (TAN), nitrite-N (NO_2-N) and nitrate-N (NO_3-N) concentration in a recirculating aquaculture system integrated with algae.

Reference	Algae biomass, g wet weight m^{-2} (g wet weight m^{-3})	NH_4-N, mg L^{-1}	NO_2-N, mg L^{-1}	NO_3-N, mg L^{-1}
**(Valeta and Verdegem, 2015)**	77.14 [1]	1.58 to 3.21	0.55 to 1.28	80.5 to 217.3
**(Huang et al., 2013)**	n.a	0.026	0.008	0.104
**(SustainAqua, 2009)**	34.72 (34.72)	negligible	negligible	negligible
**(Gál et al., 2003)**	60 [2]	0.9 to 6.02 (nitrogen)	n.a	n.a
**(Li et al., 2019)**	791[3]	0.43 ± 0.07	0.20	5.12
**(Cahill et al., 2010)**	*1230 (4098)[4]	0.03±0.01	n.a	0.03±0.01
**(Cahill et al., 2010)**	* 2430 (8111.25)[5]	0.03± 0.01	n.a	0.02±0.00
**(Pagand et al., 2000)**	2500 [6]	0.05	n.a	0.06
**(Deviller et al. 2004)**	455 to 2727[7] (909-5454)	0.34±0.12	0.13±0.05	14.5±5.1

[1] 1.08± 0.32 kg $week^{-1}$/7 day/2 m^2

[2] 2.4 to 2.6 g C m^{-2} day^{-1} (primary production)

[3] Equals to 174 mg L^{-1} TSS algae

[4] 21.64 g m^{-2} day^{-1} growth rate

[5] 34.04 g m^{-2} day^{-1} growth rate

[6] 3.3 kg.$m^{-2}$$year^{-1}$ of algal dry weight were harvested

[7] HRAP was restocked between 0.1 and 0.6 g of dry weight per litre (g DW L^{-1}) weekly in summer and autumn and biweekly in winter and spring
